# Plasma Lipid Profile and Intestinal Microflora in Pregnancy Women With Hypothyroidism and Their Correlation With Pregnancy Outcomes

**DOI:** 10.3389/fendo.2021.792536

**Published:** 2022-01-19

**Authors:** Yanjun Cai, Yajuan Xu, Yanjie Ban, Jingjing Li, Zongzong Sun, Miao Zhang, Biao Wang, Xiaofeng Hou, Yingqi Hao, Qian Ouyang, Bo Wu, Mengqi Wang, Wentao Wang

**Affiliations:** Department of Obstetrics and Gynecology, The Third Affiliated Hospital of Zhengzhou University, Zhengzhou, China

**Keywords:** hypothyroidism during pregnancy, intestinal microflora, plasma lipid metabolism, pregnancy outcomes, omics

## Abstract

**Objective:**

To investigate the lipid profiles and intestinal microflora in pregnant patients with hypothyroidism and their correlation with pregnancy outcomes.

**Methods:**

In total, 27 pregnant women with hypothyroidism (study case) and 28 normal pregnant women (control group) were enrolled in this study. The lipid profiles and intestinal microflora in the two groups were compared using untargeted liquid chromatography-mass spectrometry (LC-MS) and 16S rRNA amplicon sequencing, respectively. The association among the differential metabolites, intestinal microflora, serological indicators and pregnancy outcomes was further analyzed.

**Results:**

Patients in study case had higher C-reactive protein (CRP) levels (P = 0.025) and lower birth weight (P=0.005) than the control group. A total of 42 differential lipid metabolites and 7 enrichment KEGG pathways were obtained between the two groups (VIP ≥ 1, P < 0.05). Ten lipid metabolites can be used as characteristic metabolites of study case, including phosphatidylcholine (PC), phosphatidylethanolamine (PE) and sphingomyelin (SM). The richness and diversity of intestinal microflora in study case were lower than those in the control group (P>0.05). LEfSe analysis revealed that patients in study case had higher abundance of *Prevotella* and *Haemophilus* and lower abundance of *Blautia* than the control group (P < 0.05). *Blautia* was positively correlated with SM and negatively correlated with PC and PE; the CRP level and *Prevotella* were positively correlated; the neonatal weight and PC level were negatively correlated (P < 0.05).

**Conclusion:**

The lipid profile and intestinal microflora of pregnant women with hypothyroidism significantly differed from those of normal pregnant women and were associated with adverse pregnancy outcomes. The interaction between lipid metabolism and intestinal microflora may be a potential target for further studies investigating the pathogenesis of hypothyroidism during pregnancy.

## Introduction

Hypothyroidism in pregnancy refers to a systemic hypometabolic syndrome caused by hypothyroxinemia or thyroid hormone resistance and has a prevalence of 0.5%, and its etiology remains unclear (1). The maternal and fetal needs for thyroid hormone increase during pregnancy. If the low level of thyroid hormone in pregnant women with hypothyroidism is not effectively improved, there is an increased risk of adverse pregnancy outcomes, such as premature birth, miscarriage, and low birth weight, and the neurointelligence development of the offspring can be seriously affected (2, 3).

The intestinal microflora is an important component of the human microbiome that contributes to human physiology, metabolism, nutrition, and immune function. Disruptions to the gut microbiota have been linked to various endocrine diseases, such as diabetes, obesity, chronic metabolic disorders and Hashimoto’s thyroiditis (4, 5). Wang, Z. et al. (6) showed that the abundance of the *Paraprevotella* was increased in patients with primary hypothyroidism, whereas the abundances of the genera *Haemophilus* and *Clostridium* were decreased, and the thyroid function of mice with fecal microbiota transplantation (FMT) using flora from patients with hypothyroidism was abnormal. Our previous study also showed that the changes of oral and intestinal microbiota may be one of the factors influencing the occurrence and development of hypothyroidism during pregnancy (7). It has been reported that the intestinal microflora and metabolic disorders in pregnant mothers have important impacts on pregnant mothers and their new-borns (8). However, the relationship between plasma lipid metabolites and intestinal microflora and their effects on the pathological mechanism of hypothyroidism during pregnancy remain unclear.

Lipids are essential metabolites of organisms, which have many key cellular functions and can reflect the metabolic state of cells. The combination of Metabolomics and 16S rRNA gene sequencing is helpful to explain the close relationship between intestinal microflora and host. Previous studies have shown that host-microbiota interactions during the perinatal period impact host lipid metabolism (9). PC and other lipid metabolites can be used as potential markers for diagnosing hypothyroidism and are closely related to the occurrence and development of hypothyroidism in humans and rats (10, 11). Calabuig-Navarro et al. (12) also found that maternal obesity may play an important role in fetal growth and development by affecting lipid transport and metabolism levels in the placenta. However, the characteristics of the plasma lipid profile in patients with hypothyroidism during pregnancy are still unclear. In the current study, we used LC-MS and 16S rRNA amplification sequencing to analyze the characteristics of the plasma lipid profile and intestinal microflora in patients with hypothyroidism during pregnancy and explore the pathogenesis of hypothyroidism during pregnancy and its adverse pregnancy outcomes from a new perspective.

## Materials and Methods

### Participants

From September 2019 to April 2020, patients with hypothyroidism during the third trimester (study case, n=30) and normal pregnant women during the third trimester without hypothyroidism (control group, n = 30) were recruited in the Third Affiliated Hospital of Zhengzhou University, China. The inclusion criteria were based on the diagnostic criteria according to the 2017 Guidelines of the American Thyroid Association for the Diagnosis and Management of Thyroid Disease During Pregnancy and the Postpartum (13) and the specific threshold reference range formulated by the laboratory of the Third Affiliated Hospital of Zhengzhou University [hypothyroidism during pregnancy: FT4 < 11.5 pmol/L and TSH > 4.0 mIU/L; normal pregnancy women: 11.5 < FT4 < 22.7 pmol/L, 0.4 < TSH< 4.0 mIU/L]. The exclusion criteria included age < 18 years or > 35 years; combined endocrine diseases or immune system diseases, such as diabetes mellitus, gestational hypertension and systemic lupus erythaematosus before and during pregnancy; serious intestinal disease or history of intestinal surgery; multiple pregnancies and artificial impregnation; severe anxiety and depression; and probiotic or antibiotic treatment within the prior three months. The control group excluded volunteers with other pregnancy complications in addition to the above exclusion criteria. The two groups of pregnant women were of Han nationality who were long-term residents of Henan Province and had a similar dietary structure. Three patients in the study case and two pregnant women in the control group were excluded due to the lack of plasma specimens; therefore, 55 subjects (n =27 and n = 28, study case vs. control group) were ultimately included in the follow-up analysis. This study has been approved by the Ethics Committee of the Third Affiliated Hospital of Zhengzhou University, and each subject voluntarily signed an informed consent before the study.

### Specimen Collection

The two groups of pregnant women were in the third trimester (gestational age between 28 weeks to 41weeks). Among them, the pregnant women in the study case were first diagnosed with hypothyroidism when they visited our hospital during the third trimester of pregnancy, and their fecal and plasma samples were collected on the day of enrollment. On the morning of the day of enrollment, approximately 5ml of fasting peripheral venous blood was collected from all participants. After collection, the blood specimens were immediately placed in a refrigerator at 4°C for temporary storage and then centrifuged at 1600 g for 10 min at 4°C within 2 h after collection. The upper layer, i.e., plasma, was collected and frozen at -80°C. Fecal samples were obtained from all participants on the day of enrollment. The subjects were asked to provide approximately 5-100 mg of fresh stool in a 2.0 ml cryogenic tube using a sterile spoon. The specimens were transported to the laboratory in dry ice within 2 h and stored at -80°C.

### Untargeted Metabolomics Analysis

Lipid molecules were extracted from the specimens. Untargeted lipidomics samples were analyzed in the positive and negative ionization mode using an ultra-performance liquid chromatography system (Waters 2D UPLC, Waters, USA) and a high-resolution mass spectrometry system (Q Exactive, Thermo Fisher Scientific, USA). The raw data were imported into LipidSearch v.4.1 (Thermo Fisher Scientific, USA) for peak alignment. To normalize the data, lipid molecules that were not observed in 80% of the samples were omitted from the final analysis. Lipid molecules with a relative peak area greater than 30% based on the coefficient of variation were omitted from all QC sample analyses (QC quality control samples were obtained by mixing 10 μL of supernatant from each sample). A univariable method [fold change (FC) and T test] was used to analyze the metabolites with large differences in abundance among the different subgroups. Then, the peak table was imported into the self-developed metabolomics R software package metaX (14) to obtain clustering information and identify variables with significant differences between the experimental and control groups. Then, a partial least squares-discriminant analysis (PLS-DA) model was established. In the PLS-DA model, with seven cross-validations, the differential lipid molecules were selected as biomarkers if the variable importance (VIP) values of the first two principal components were ≥ 1 and the FC in variance was ≥ 1.2 or ≤ 0.83; the model was tested with P < 0.05 at the univariate level after correction. The KEGG metabolic pathways of the differentially expressed metabolites were identified.

### Sequencing and Data Analysis of the V3-V4 Region of 16S rRNA

Total DNA was extracted from bacterial populations in the stool specimens (0.20 g) using a KF Kit B (Magen, China). Double-index fusion primers containing sequencing adaptors were designed and synthesized. The V3-V4 hypervariable region of the bacterial 16S rRNA gene was amplified using polymerase chain reaction (PCR). The PCR products were purified, and the quality was verified using the magnetic beads method; then, a qualified sequencing library was constructed. The subsequent sequencing was performed using the Illumina MiSeq platform (BGI, Shenzhen, China) (15), which generated 2×300 bp paired-end reads. After the offline data were filtered, the overlapping relationship, which was determined using FLASH (Fast Length Adjustment of SHort reads, v1.2.11) (16) software, was used to stitch pairs of reads into hypervariable region tags. Tag clustering was performed using 97% similarity to obtain operational taxonomic units (OTUs), followed by species annotation. Alpha diversity analyses (Chao index, ACE index, Shannon’s diversity index, Simpson’s diversity index, and Good’s diversity index), beta diversity analyses (Bray-Curtis analysis, unweighted UniFrac analysis, and weighed UniFrac analysis), and intergroup significant difference analyses (LEfSe software) were performed based on the OTU and species annotation results. Then, PICRUST software (17) was used to predict the Kyoto Encyclopedia of Genes and Genomes (KEGG) pathways and COG functions of flora, and the results were visualized by R language permutation.

### Data Collection

We obtained gestational week, body mass index (BMI), CRP, TSH, FT4 and thyroid peroxidase antibody (TPOAb) levels at the time of collection of fecal and plasma samples. Subsequently, patients in the study case were given oral levothyroxine tablets according to the guidelines (13), and TSH, FT4 indicators were recorded before delivery. In both groups, we also recorded clinical information at the time of delivery, such as the blood loss, placental weight, neonatal weight, head circumference, and body length.

### Statistical Methods

The normally distributed quantitative data were described as means ± standard deviations, the non-normally distributed data were described by medians and quartiles, and the comparisons between groups were described by T tests, Wilcoxon rank sum tests. The SPSS software (version 26.0) was used for analysis. A double-tailed P of < 0.05 indicates statistically significant difference. The correlations between the variables (clinical parameters, differential intestinal microflora, and plasma metabolites) were calculated using a Spearman rank correlation analysis, and the correlation coefficients were obtained using a network model (Cytoscape, Version 3.5.1) (t test, p < 0.05, | correlation coefficient | > 0.3).

## Results

### Comparison of Baseline Data Between the Two Groups

No significant difference was found in age, BMI and gestational week between the study case and control group (P > 0.05), which minimized the influence of confounding factors on the experimental results. The serum CRP levels in the study case were significantly higher than those in the control group (P = 0.025), and the neonatal weight and length were significantly lower than those in the control group (P=0.005, P< 0.001, **Table 1**).

**Table 1:** Characteristics of pregnant women with hypothyroidism and the control group.

Parameters	Case H	Case C	p Value
Age^#^, (years)	29.00 (28.00,32.00)	30.50 (28.00,32.00)	0.886
BMI^*^, (kg/m^2^)	26.15 ± 3.28	26.83 ± 3.35	0.451
Gestational weeks^#^, (weeks)	36.43 (33.29,37.86)	37.14 (35.32,38.14)	0.316
FT4 ^#^, (pmol/L, at time of sampling)	10.10 (9.70,10.70)	12.25 (11.70,13.33)	**0.001**
TSH^#^, (mIU/L, at time of sampling)	4.53 (4.23,5.60)	1.86 (1.06,2.58)	**0.001**
FT4, (pmol/L, after treatment)	9.94 ± 1.26	–	**-**
TSH, (mIU/L, after treatment)	3.24 ± 1.52	–	**-**
TPOAb^#^, (U/mL)	9.96 (9.47,10.70)	8.42 (7.88,9.12)	**0.001**
CRP^#^, (mg/L)	4.00 (2.00,13.00)	2.50 (1.25,5.00)	**0.025**
Head circumference of newborn^#^, (cm)	33.00 (32.00,34.00)	35.00 (35.00,36.00)	**0.001**
Body length of newborn^#^, (cm)	50.00 (49.00,50.00)	52.00 (51.00,52.00)	**0.001**
Body weight of newborn ^*^, (kg)	3.19 ± 0.32	3.45 ± 0.33	**0.005**

Boldface indicates statistical significance. *Data are expressed as means ± standard deviation. ^#^Data are expressed as median (P25, P75).

### Differential Plasma Lipid Metabolites and KEGG Pathways in the Two Groups

**Figure 1:**
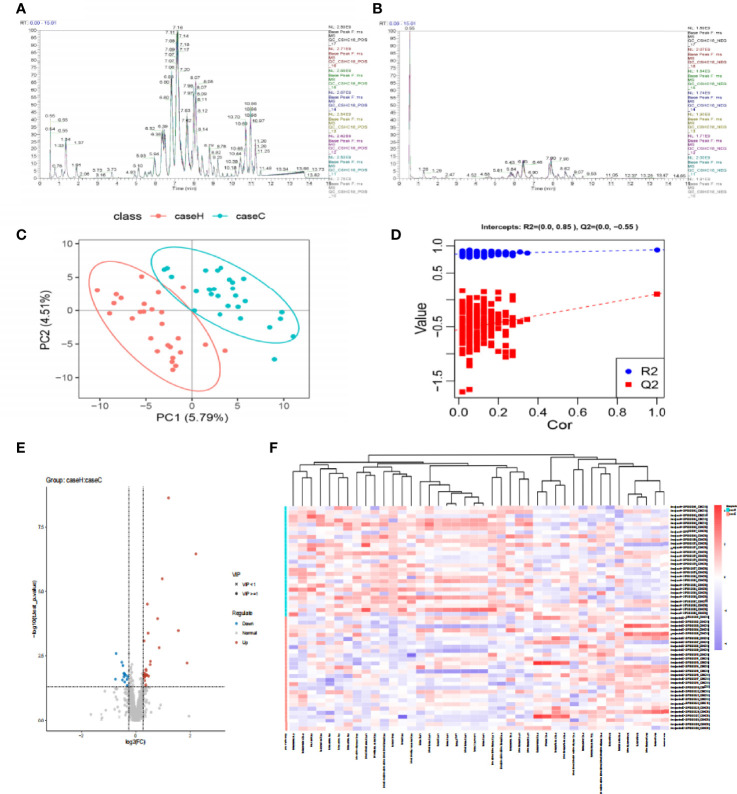
The difference of lipid metabolites between the two groups. **(A, B)** The Based Peak Chromatogram (BPC) overlap plot of the QC samples in the anionic and cationic modes. **(C)** PLS-DA score plot shows a significant difference between the two groups (2 principal components, PC1 score: 5.79%, PC2 score: 4.51%). The distance of each coordinate point represents the degree of aggregation and dispersion between samples. **(D)** Model verification map of PLS-DA intercepts: R2 = (0.0, 0.85), Q2 = (0.0, −0.55). **(E)** Volcanic map of differential metabolites. The abscissa is the multiple change value of the expression difference of metabolites between the two groups, and the ordinate is the statistical test value of the expression difference of metabolites, that is, p-value. The abscissa and ordinate values are all logarithmically processed. Round is lipid molecules with VIP≥1, “×” is lipid molecules with VIP<1, blue is down-regulated, red is up-regulated in the study case. **(F)** The hierarchical cluster heatmap of differential metabolites. The columns represent differential metabolites, the rows represent samples and. The color from blue to red indicates expression abundance of metabolites from low to high.

The base peak chromatogram (BPC) of all QC samples in the anionic and cationic modes are shown in **Figures 1A, B**, and the spectra overlapped well, indicating that the instrument was in good condition and the detection results were reliable. PLS-DA (**Figure 1C**) and model validation (**Figure 1D**) revealed the study case and control group could be separated into distinct clusters according to their metabolic differences (R2Y = 0.85, Q2 = -0.55). Based on the analysis results of PLS-DA and using VIP≥1 and P < 0.05 as the screening criteria, we screened out a total of 42 differential lipid metabolites (14 downregulated and 28 upregulated) in the study case (**Figure 1E** and **Table S1**). To evaluate the rationality of the candidate metabolites and more fully and intuitively illustrate the differences in expression of metabolites between the study case and control group, we conducted a hierarchical cluster analysis based on the expression of significantly different metabolites in each group of samples (**Figure 1F**), which showed that most of the metabolites involved in glycerophospholipid metabolism were up-regulated in the study case, including P-Choline, P-Ethanol Amine and some P-Inositol, while the down-regulated metabolites included some sphingolipid metabolism (Sphingomyelin).

**Figure 2:**
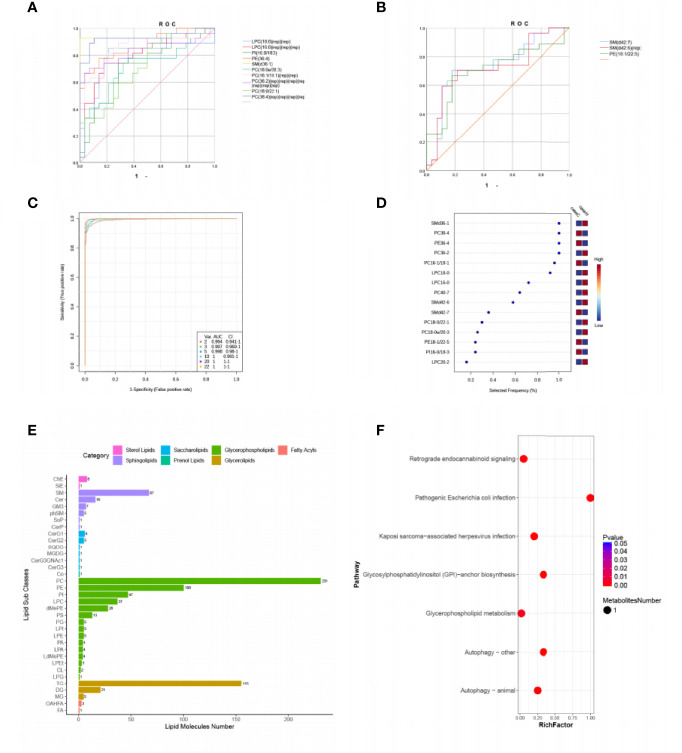
Comparison of variables based on ROC curve and KEGG pathway analysis for differential metabolites. **(A, B)** ROC curves of 22 differential metabolites (VIP ≥ 2, P < 0.05, Area under the curve (AUC) > 0.7). **(C)** Multivariate ROC based exploratory analysis, showing the feature numbers, the AUCs and the confidence intervals of the six models. **(D)** The percentage selected frequency of metabolites based on ROC curves, with the VIP plot indicating the most discriminating metabolite in descending order of importance. **(E)** The ordinate shows the lipid subclasses identified in this experiment. The abscissa is the number of lipid molecules identified in each lipid subclass, and the different colors indicate the different lipid classes. **(F)** KEGG pathways on level 3 related to 42 differential metabolites. The ordinate is the name of pathway level 3, and the abscissa is the ratio of the number of differential metabolites enriched in the pathway to the number of metabolites annotated in the pathway. The size and color of bubbles represent the number and degree of enrichment of different metabolites, respectively.

Then we focused on top 22 differential metabolites with VIP≥2 and P < 0.05 (**Table 2**), calculated their area under the curve (AUC) by using the receiver operating characteristic (ROC) curves (**Figures 2A** **, B**). Multivariate ROC curve based exploratory analysis was tried to achieve a better predictive model using these 22 combined differential metabolites. A panel consisting of SM(d36:1), PC(38:4), PE(36:4), PC(36:2), PC(16:1/18:1), LPC(18:0), LPC(16:0), PC(40:7), SM(d42:6), SM(d42:7) showed the best predictive ability with ROC area of 1 for the testing dataset (AUC = 1; 95% CI, 0.991–1.00; **Figures 2C** **, D**). These ten metabolites were identified as characteristic metabolites for patients with hypothyroidism during pregnancy. We further analyzed 796 lipid metabolites identified in this experiment and found that most of them belong to Glycerophospholipid metabolites (**Figure 2E**). To identify the most relevant metabolic pathways involved in hypothyroidism during pregnancy, we used the KEGG enrichment analysis to analyze the pathways of the metabolites that differed between the two groups. Metabolic pathways involving autophagy, glycerophospholipid metabolism, etc. were highlighted as targets for investigating the pathological mechanisms underlying hypothyroidism during pregnancy (p < 0.01, **Figure 2F**).

**Table 2:** Relative abundance of 22 differential metabolites (Mean with SD).

Lipid	RT	CalcMz	Case C	Case H	VIP	FC	*p* value	Regulate
LdMePE(18:2)	1.08	504.31	1421887.31 ± 467125.2	1843541.18 ± 718075.7	1.92	1.3	0.02	Up
LPE(18:2)	1.17	478.29	18635655.28 ± 9223.37	24153303.58 ± 9223.37	1.99	1.3	0.01	Up
LPC(15:2)	1.18	478.29	18431978.83 ± 9223.37	23814565.8 ± 9223.37	2	1.29	0.01	Up
LPC(20:2)	1.5	548.37	18246811.85 ± 9223.37	23090200.07 ± 9223.37	1.96	1.27	0.02	Up
LPC(18:0)	2.17	524.37	19336883.32 ± 12184359.87	57053312.82 ± 66136289.14	3.87	2.95	0.001	Up
LPC(16:0)	2.18	496.34	9223.37 ± 9223.37	9223.37 ± 9223.37	3.18	1.74	0.001	Up
PI(34:3)	3.84	831.5	348352.14 ± 174548.01	499951.14 ± 237632.2	2.21	1.44	0.01	Up
SM(d42:7)	4.75	847.6	599416.34 ± 283357.94	417588.66 ± 178321.33	2.12	0.7	0.01	Down
PI(36:3)	5.38	859.53	2430344.23 ± 1313209.51	3204349.61 ± 1235383	2.32	1.32	0.02	Up
PE(36:5p)	5.81	720.5	1168646.26 ± 971419.59	691770.58 ± 273689.03	2.11	0.59	0.03	Down
PE(36:4)	6.48	740.52	10453406.69 ± 9223.37	20485685.7 ± 9223.37	3.63	1.96	0.001	Up
SM(d42:6)	6.6	805.62	24713435.77 ± 11527622.05	17691592.53 ± 9223.37	1.98	0.72	0.01	Down
PE(40:6)	6.82	790.54	830027.58 ± 468597.41	499096.38 ± 514640.18	3.99	0.6	0.001	Down
PC(36:5)	7.05	780.55	410405627.11 ± 162257348.95	306783361.22 ± 173603056.82	2.12	0.75	0.02	Down
PC(40:7)	7.45	832.59	9223.37 ± 9223.37	9223.37 ± 9223.37	5.03	1.81	0.001	Up
PC(40:5)	7.46	880.61	13071670.39 ± 9223.37	9223.37 ± 9223.37	2.26	0.74	0.02	Down
SM(d36:1)	7.8	731.61	82510012.76 ± 71008351.74	382298802.52 ± 127825514.29	6.98	4.63	0.001	Up
PC(38:3e)	8.11	842.63	9223.37 ± 9223.37	10303669.8 ± 9223.37	1.99	1.44	0.01	Up
PC(34:2)	8.18	758.57	115959858.12 ± 22606080.86	157881110.87 ± 41723679.71	2.51	1.36	0.001	Up
PC(36:2)	8.45	808.58	31633500.55 ± 9223.37	42373970.06 ± 9223.37	2.29	1.34	0.001	Up
PC(40:1)	9.16	888.67	113609.85 ± 92233.72	418552.01 ± 92233.72	3.98	3.68	0.01	Up
PC(38:4)	9.68	810.6	9223.37 ± 9223.37	11916391.68 ± 9223.37	5.47	2.3	0.001	Up

RT, Retention time; VIP, variable important in projection; FC, fold change; up, the expression of metabolite in study group increased; down, the expression of metabolite in study group decreased.

### Characteristics of Intestinal Microflora Between the Two Groups

**Figure 3:**
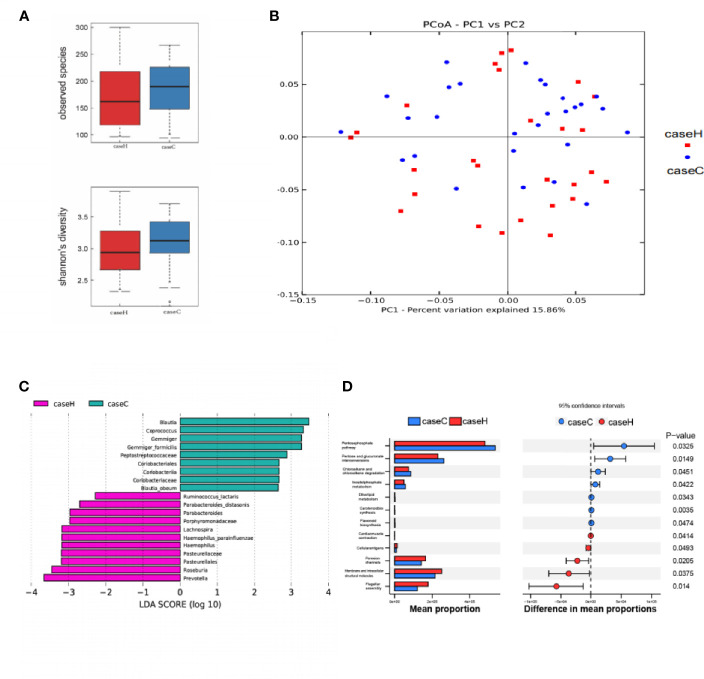
Characteristics of intestinal microflora between the two groups. **(A)** The Sobs index and Shannon index of the study case was lower than that of control group, while no significant difference was found between the two groups. **(B)** PCoA of intestinal microflora by Bray–Curtis distance. **(C)** LDA chart. The score was obtained by Lefse analysis. The control group-enriched taxa are indicated by a positive LDA score (green), and the study case-enriched taxa are indicated by a negative LDA score (pink). The greater the LDA score, the greater the impact of the representative species abundance on the differences between the two groups. Only taxa meeting a significant LDA score > 2 are shown. **(D)** Predicted functions of intestinal microflora by PICRUSt. On the left is a bar chart showing species abundance differences between the two groups. On the right is the dot bar plot, which shows the percentage of all species in each sample between the two groups. Red is the study case, blue is the control group (p < 0.05).

Compared with the control group, the study case showed lower Sobs index and lower Shannon index with no statistically significant difference (P > 0.05, **Figure 3A** and **Table S2**). These results suggested that the richness and diversity of intestinal microflora in the study case were lower than those of the control group. PCoA by Bray–Curtis distance showed no significant difference between the two groups (**Figure 3B**). LEfSe analysis showed that among the top 20 genera with significant difference, *Prevotella, Haemophilus, Roseburia*, etc. were significantly higher in abundance in the study case compared with the control group; *Blautia, Coprococcus, Gemmiger*, etc. showed significantly lower abundance in the study case compared with the control group. LDA showed that *Prevotella, Blautia* were the important characteristic genera (**Figure 3C**). PICRUST software was used to predict the KEGG pathways of the intestinal microflora in the two groups, and significant differences were found in 12 functional pathways at the L3 level, including etherlipidmetabolism, Inositolphosphatemetabolism pathway, etc. (P < 0.05, **Figure 3D**).

### Correlation of Differential Lipid Metabolites with Intestinal Microflora, Clinical Indicators and Pregnancy Outcomes

**Figure 4:**
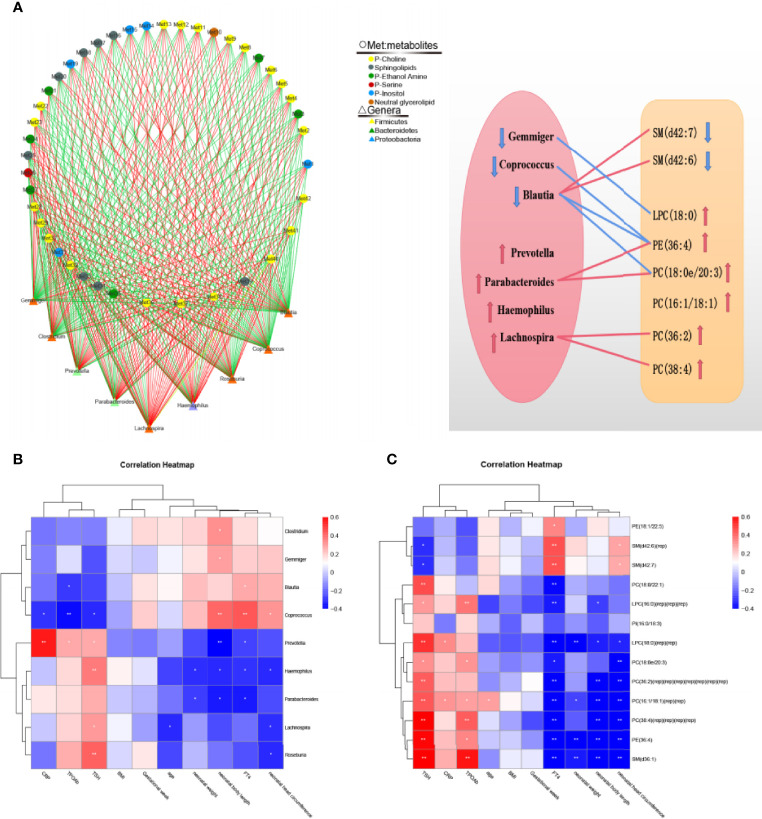
Correlation analysis of intestinal microflora, lipid metabolites, serological indicators and pregnancy outcomes. **(A)** Correlation network diagram between 42 differential metabolites and the top 9 genera in abundance. Negative correlation are indicated as green edges, and positive correlation are indicated as red edges. The correlation map shows their connection intuitively. The red line represents a significant positive correlation, while the blue line represents a significant negative correlation; the up arrow represents an increase in abundance, while the down arrow represents a decrease in abundance. **(B)** Correlation heatmap between serological indicators, pregnancy outcomes and the top 9 genera in abundance. **(C)** Correlation heatmap between serological indicators, pregnancy outcomes and 13 important differential lipid metabolites. The color intensity represents magnitude of correlation. Red is positive correlations, blue is negative correlations, *p < 0.05, **p < 0.01.

Through Spearman’s correlation analysis, the correlations among lipid metabolites, intestinal microflora, clinical indicators and pregnancy outcomes were reviewed. The correlation analysis of 42 different lipid metabolites and 9 different genera showed that the abundance of *Blautia* positively correlated with that of SM (d42:6) and SM (d42:7) and negatively correlated with that of PE(36:4) and PC(16:1/18:1) (P < 0.05). On the basis of the relationship between lipid metabolites and intestinal microflora, we drew a correlation network and a correlation map to show their connection intuitively (**Figure 4A** and **Table S3**). The correlation analysis among clinical indicators, pregnancy outcomes and intestinal microflora showed that the levels of serum CRP and TSH positively correlated with the abundance of *Prevotella*; the levels of serum FT4 positively correlated with the abundance of *Blautia*; and the neonatal weight and length negatively correlated with the abundance of *Haemophilus* (**Figure 4B**). The correlation analysis between pregnancy outcomes and lipid metabolites showed that the neonatal weight and length negatively correlated with the abundance of PC (16:1/18:1), LPC (18:0) and PE (36:4) (**Figure 4C**).

## Discussion

Hypothyroidism is a common complication of pregnancy, and its incidence is increasing yearly (1), which can lead to a series of short- and long-term maternal and fetal complications (2). Currently, the pathogenesis of hypothyroidism during pregnancy is still unclear. With the application of multiomics techniques, increasing evidence indicates that intestinal microflora may play an important role in the occurrence and development of thyroid disease by destroying the intestinal barrier function and leading to immune inflammation and metabolic disorder (18).

In this study, we revealed the differences in plasma lipid metabolites, intestinal microflora, clinical indicators and pregnancy outcomes between the patients with hypothyroidism during pregnancy and the control group and discussed the correlation among the four factors. In general, according to the metabolomic profile analysis, we found the important KEGG enrichment pathways and determined 10 characteristic metabolites, which may be used to distinguish hypothyroidism during pregnancy. The 16S rRNA analysis illustrated that the composition and structure of intestinal microflora were significantly different between the two groups. Moreover, correlation analysis revealed a close association among the important differential lipid metabolites and intestinal microflora, clinical indicators and pregnancy outcomes in the study case, which may provide hints to investigate hypothyroidism during pregnancy pathogenesis.

We successfully screened 10 differential metabolites that could be used as characteristic metabolites for patients with hypothyroidism during pregnancy by untargeted metabolomics analysis, including increased levels of PC (36:2), PE(36:4) and decreased levels of SM(d42:6), SM(d42:7), etc. Liu, J. et al (19). found that compared with the control group, the levels of PC and PE in patients with hypothyroidism were increased, which is consistent with our results. PC is a lipid metabolite necessary for assembly and secretion of low density lipoprotein-cholesterol (LDLC) (20). Indeed, increased serum levels of triglycerides and LDLC have long been considered to have direct effects on hypothyroidism (21). Similar research also reported that increased serum levels of LDLC could increase the risk of hypothyroidism in women by approximately one times compared with women with normal levels of LDLC (22). The Increased levels of PC may be associated with thyroid dysfunction by affecting LDLC levels. In addition, our KEGG pathway analysis in the study case demonstrated that PE couples with microtubule-associated protein light chain 3 (LC3) to form membrane-bound LC3-PE, which plays an important role in autophagy pathway, suggesting that PE may play an important role in the immunoinflammatory response of hypothyroidism during pregnancy (23).

Changes in the lipid metabolites, such as glycerophos-pholipids and sphingolipids have been reported to have a close relationship with intestinal microflora (24). Thus, we further analyzed the characteristics of intestinal microflora in the two groups. Compared with the control group, although the alpha and beta diversity were not statistically significant, the diversity and richness of the intestinal microbiota were lower in the study case, which is consistent with our previous study (7). LEfSe analysis showed an increased abundance of *Prevotella* and *Haemophilus* and a decreased abundance of *Blautia* in the study case. Wang, Z. et al (6). found that the alpha diversity of the intestinal microflora and the abundance of the *Paraprevotella* were increased, while the abundances of *Haemophilus* and *Clostridium* were decreased in patients with primary hypothyroidism. The causes of the differences in the alpha diversity and intestinal microflora between our and their studies are unclear, likely due to the complicated interaction among the intestinal microflora. In addition, pregnancy as a special period of life, the composition of intestinal microflora of them may be different from that of normal people due to significant changes in maternal hormone levels. *Prevotella* and *Haemophilus* are floras belonging to Gram-negative bacteria and settle normally in the human gastrointestinal tract; its disorder can cause Hashimoto’s Thyroiditis, inflammatory bowel disease and other diseases (25, 26). The increased abundance of *Prevotella* and *Haemophilus* in hypothyroidism during pregnancy may be related to its characteristics in autoimmune process. The autoimmune processes can recognize and destroy normal thyroid follicular cells, affecting thyroid hormone synthesis and resulting in hypothyroidism. *Blautia* could produce SCFAs. SCFAs, as the energy sources of enterocytes, could maintain the intestinal epithelial barrier, decrease the permeability of the gut and reduce inflammation *via* GPR41/43 (27). Thus, we speculate that the decreased abundance of *Blautia* is related to the increased intestinal permeability of patients with hypothyroidism during pregnancy.

Correlation analysis revealed a close relationship between intestinal microflora and characteristic plasma lipid metabolites. We found that the abundance of *Blautia* positively correlated with the abundance of plasma SM(d42:6) and SM(d42:7) and negatively correlated with the abundance of plasma PC (38:3e) and PE (36:4). PC and PE are important components of biofilm, that belong to glycerophospholipids metabolites. Li, Z. et al. (28). researched on the Pemt (-/-) mice fed a choline-deficient (CD) diet and showed that the ratio of PC/PE levels is a key regulator of cell membrane integrity and plays a role in the progression of steatosis into steatohepatitis. The uptake and utilization of thyroid hormone may be affected when the integrity of the biofilm is destroyed. In addition, PC is also involved in the lipopolysaccharide (LPS)-induced proinflammatory response of macrophages (29). Hypothyroidism during pregnancy has been proved to be an immune-metabolic disease, and dyslipidemia are common symptoms (21, 30). In thyroid cells, the proinflammatory factor-induced conversion of SM to ceramide is considered a critical signaling pathway involved in apoptosis, and the conversion of SM to ceramide can competitively inhibit the activity of type 1 iodothyronine deiodinase (Dl) (31, 32). Therefore, the decrease in SM may be related to the destruction of thyroid follicular cells and the disturbance in thyroid hormone synthesis. However, this study has not detected a difference in the level of ceramide between the two groups, likely due to the inability to detect low concentrations or metabolite detection platform is unsuitable. Consistent with our research, Feng J. et al. (33) studied thyroid cancer patients and found that *Blautia* was closely related to the plasma SM levels. The predicted KEGG pathways that were closely correlated with *Blautia* in our study case, include the etherlipidmetabolism and Inositolphosphatemetabolism pathways. Therefore, we hypothesize that intestinal microflora dysbiosis is closely correlated with hypothyroidism during pregnancy and that glycerophospholipid and sphingolipid metabolism metabolism dysregulation might be a crucial factor.

In addition, correlation analysis revealed a linear relationship between the clinical serological indexes and intestinal microflora, that is, the levels of serum CRP and TSH positively correlated with *Prevotella*. CRP, as a nonspecific marker of inflammation and tissue damage, its increased levels suggest an increased pro-inflammatory response. However, the gram-negative bacteria *Prevotella* can induce the transformation of Type 1 T helper cells (Th1) to Type 2 T helper cells (Th2) through lipopolysaccharide (LPS) on their outer membrane to activate the intestinal immune inflammatory system and then destroy the intestinal barrier to cause a “leaky gut” (34, 35), triggering a congenital autoimmune response against thyroid cells (36), which may lead to a reduction in serum thyroid hormone levels and increase TSH levels by negative feedback mechanisms, thereby leading to hypothyroidism. Moreover, we found a significant positive correlation between serum FT4 level and the abundance of *Blautia*. *Blautia* is an anti-inflammatory bacterium whose metabolite, SCFAs, can directly affect the synthesis and utilization of thyroid hormones and participate in the maintenance of thyroid hormone homeostasis (37, 38). Therefore, we speculate that *Prevotella* and *Blautia* may be involved in maintaining the balance of thyroid hormone through immune inflammation and metabolism, which may be related to the pathogenesis of hypothyroidism during pregnancy.

In this study, the neonatal body weight and length in the study case were significantly lower than that in the control group and were negatively correlated with plasma PC (16:1/18:1), LPC (18:0) and PE (36:4) levels. PC, LPC and PE belong to glycerophospholipid metabolites, which have been proved to be closely related to metabolic disorder in hypothyroidism patients and animal models (19, 39). Stojanovska et al. (40, 41). showed that maternal plasma lipid metabolism disorders, especially increases in PC levels, can cause fetal growth restriction and significantly affect neonatal lipid metabolism, which is consistent with the results of this study, suggesting that PC and other lipid metabolites may play an important role in the adverse pregnancy outcomes of patients with hypothyroidism during pregnancy. On the one hand, PC is a major membrane phospholipid that can increase the release of proinflammatory cytokines, such as interleukin-6 (IL-6) and tumor necrosis factor-α (TNF-α). Proinflammatory reactions and further accumulation of syncytiotrophoblasts on the outer surface of placental villi will then lead to the activation of macrophages, interfere with the homeostasis of the placental barrier and affect the exchange of fetal-maternal metabolites, thereby affecting neonatal body weight and length. On the other hand, the gram-negative bacteria *Haemophilus* can cause LPS to cross the placental barrier and activate inflammatory factors (TNF-α) disrupting the vascular endothelial barrier, thereby affecting the placental blood supply and leading to a range of adverse pregnancy outcomes (42). In this study, the results of the negative correlation between *Haemophilus* and newborn weight may prove this point. However, the specific reasons for this result are unclear and require further investigation.

The advantages of this study are as follows: (1) maternal fecal and plasma samples and their serological indicators were collected simultaneously to explore the relationship among the intestinal flora, plasma lipid metabolites and host immune metabolic function within a minimum time interval, and (2) the clinical characteristics related to pregnancy outcomes were included to explore the effects of the intestinal flora and lipid metabolites on maternal and infant outcomes. However, there are still some limitations in this study as follows: (1) the sample size is small, which may lead to bias in the results, and (2) to eliminate the effect of levothyroxine sodium tablets treatment on maternal gut flora and lipid metabolism, this study only included pregnant women who were diagnosed with hypothyroidism in the third trimester due to poor perinatal health care in the first and second trimesters of pregnancy of pregnancy; thus, it was not possible to study the dynamic changes in gut flora and lipid metabolism throughout pregnancy. Therefore, it is necessary to design longitudinal studies with larger sample sizes using animal models and multigroup analysis techniques to further reveal the mechanism underlying hypothyroidism in pregnancy.

In summary, this study analyzed the changes in plasma lipid metabolites and intestinal microflora in patients with hypothyroidism during pregnancy. It was found that ten lipid metabolites, such as PC, PE and SM can be used as characteristic metabolites for patients with hypothyroidism during pregnancy. In these patients, lipid metabolites, intestinal microflora, clinical indicators and pregnancy outcomes were closely correlated, and the characteristic lipid metabolites and intestinal microflora may play a role in the occurrence and development of hypothyroidism during pregnancy and adverse pregnancy outcomes through interactions with immune inflammatory reactions. The relationship among SM, PC and *Blautia* may provide new evidence for studies investigating the pathogenesis of hypothyroidism during pregnancy.

## Data Availability Statement

The original contributions presented in the study are included in the article/**Supplementary Material**. Further inquiries can be directed to the corresponding author.

## Author Contributions

Conceptualization, YC and YX. Methodology, YC. Software, YB. Validation, YC, JL, ZS. Formal analysis, YC. Investigation, ZS. Resources, MZ, YH, QO. Data curation, XH, BoW. Writing—original draft preparation, YC, YX. Writing—review and editing, YX. Visualization, BiW. Supervision, ZS. Project administration, YB. Funding acquisition, MW, WW. All authors have read and agreed to the published version of the manuscript.

## Funding

This work was supported by Henan provincial science and technology research and development special funds 182102410020409 (YjX).

## Conflict of Interest

The authors declare that the research was conducted in the absence of any commercial or financial relationships that could be construed as a potential conflict of interest.

## Publisher’s Note

All claims expressed in this article are solely those of the authors and do not necessarily represent those of their affiliated organizations, or those of the publisher, the editors and the reviewers. Any product that may be evaluated in this article, or claim that may be made by its manufacturer, is not guaranteed or endorsed by the publisher.
